# Modeling Microbial Community Networks: Methods and Tools for Studying Microbial Interactions

**DOI:** 10.1007/s00248-024-02370-7

**Published:** 2024-04-08

**Authors:** Shanchana Srinivasan, Apoorva Jnana, Thokur Sreepathy Murali

**Affiliations:** Department of Public Health Genomics, Manipal School of Life Sciences, Manipal Academy of Higher Education, Manipal, 576104 India

**Keywords:** Microbiome, Microbial interactions, Network inference, Dynamic modeling

## Abstract

Microbial interactions function as a fundamental unit in complex ecosystems. By characterizing the type of interaction (positive, negative, neutral) occurring in these dynamic systems, one can begin to unravel the role played by the microbial species. Towards this, various methods have been developed to decipher the function of the microbial communities. The current review focuses on the various qualitative and quantitative methods that currently exist to study microbial interactions. Qualitative methods such as co-culturing experiments are visualized using microscopy-based techniques and are combined with data obtained from multi-omics technologies (metagenomics, metabolomics, metatranscriptomics). Quantitative methods include the construction of networks and network inference, computational models, and development of synthetic microbial consortia. These methods provide a valuable clue on various roles played by interacting partners, as well as possible solutions to overcome pathogenic microbes that can cause life-threatening infections in susceptible hosts. Studying the microbial interactions will further our understanding of complex less-studied ecosystems and enable design of effective frameworks for treatment of infectious diseases.

## Introduction

Microbial communities are critical to the functioning of various ecosystems and critically impact nutrient cycling, agriculture, health, and disease [1]. These communities exhibit a variety of emergent behaviors such as biofilm formation and quorum sensing as a consequence of several inter/intra-species interactions that range from competition for nutrient sources to cooperative networks coordinated by metabolite exchange [2, 3]. An understanding of the nature of microbial interactions can help us better understand mechanisms of their stability and function [4]. However, dynamic and complex interactions within microbial communities can rarely be classified under one category. This brings forth the question: why is it vital to characterize and profile the interactions within the microbial consortia? At the microscale level, the primitive microbial multicellular structures display remarkable spatial structure and malleability to external stimuli. In a spatially structured community, cooperative behaviors are prevalent due to increased frequency of interactions between cells of same genotype. Spatially unstructured or well-mixed communities predominantly exhibit competition [5]. Arrangement of microbes in monospecies and mixed co-cultures is driven by their social behavior which ultimately alters growth and survival, gene expression, and physiology of individual cells. This would further impact the community’s social dynamics and structure, which in turn vastly influences its functional landscape [6].

Microbial interactions can be classified as positive, negative, or neutral based on its impact on the microbes involved [7]. Positive interactions are defined as those wherein at least one of the partners is benefited as a direct consequence of the interaction. A well-known example of cooperative behavior is quorum sensing that allows for synchronized gene expression coordinated by release of diffusible signaling molecules sensed by bacterial quorum [8]. Positive interactions wherein both partners benefit either by sharing nutrients, enzymes, and/or metabolites are described as mutualism or symbiosis. An example is the beneficial metabolic exchange between mycorrhizal fungus *Laccaria bicolour* and bacterium *Pseudomonas aeruginosa* [9]. *P*. *aeruginosa* contributes thiamine for fungi growth while *L*. *bicolour* releases trehalose, a vital chemoattractant for *P*. *aeruginosa*. Commensalism is a positive interaction where one partner benefits while the other remains unaffected. Mathis and Bronstein [10] argued that commensalism cannot be defined as a single type of interaction and discussed two possible interactions from the viewpoint of the unaffected partner; a “no-effects commensalism” where the partner gains neither benefit nor cost from the interaction and a “balanced-costs-and-benefits commensalism” wherein a partner might gain both benefits and costs but the net effect being zero. Both forms of positive interactions, bidirectional mutualism and unidirectional commensalism serve to increase a community’s productivity albeit with a decrease in stability [11]. Negative interactions are one where one microbial population negatively affect another microbial population. The causes of negative interactions can vary from competition for shared resources, production of toxic byproducts to sequestration of metabolites. Competitive behaviors have been found to be crucial to community assembly in soil ecosystems allowing for niche segregation that prevents infiltration of rare communities [12]. Parasitism, wherein one partner experiences costs and benefits at the disadvantage of the other has been best illustrated in the gut microbiome. Commonly reported gut parasites such as *Entamoeba histolytica*, *Giardia intestinalis*, and *Tritrichomonas suis* cause host damage by producing mucolytic enzymes that degrade mucins (glycosylated macromolecules) present in epithelial barrier of the gut thereby facilitating their entry into the host cells while causing extensive damage to the host [13]. Amensalism is a type of negative interaction wherein one partner causes harm to the other while receiving no harm or benefit. These interactions are often observed in metabolic networks wherein one partner’s metabolite affects the other with no benefit or harm to the former. An example is the interaction of *Saccharomyces cerevisiae* with *Oenococcus oeni* in wine wherein *S. cerevisiae* produces ethanol as a fermentation byproduct which harms *O. oeni* by interfering with its genes encoding cell wall, membrane biogenesis, and metabolite transport [14]. Other mechanisms of inhibition commonly employed in negative interactions are siderophore production (depletes partner of essential nutrient such as iron), antibiotic production (direct cell killing), or quorum sensing inhibition [15]. The study of microbial interactions has often focused on inhibitory interactions to aid in the development of antimicrobials. The focus is gradually shifting towards gaining a mechanistic understanding of the structure and behavior of all dynamic interactions [16].

Microbial interactions can be understood by deciphering the signaling molecules/metabolites exchanged, shared or cross-fed among the microbial partners and their ecological outcomes. They can be inferred via classical microbiology methods such as co-culturing the microbes together and measuring the desired metabolites, treating microbes with supernatant, extracellular vesicles or specific proteins and metabolites derived from the interacting microbial partners and/or via fluorescence labeling [17–19]. These methods are broadly classified as qualitative methods. With the advent of high throughput molecular technologies, the interaction between microorganisms is beginning to be elucidated at an unprecedented level. Data obtained with technologies such as metagenomics, transcriptomics, proteomics, and metabolomics coupled with qualitative experiments [20, 21] can provide information even on unculturable microbes, which accounts for the majority of microbes currently known. The combined data from these methods can be fed into mathematical equations to form microbial networks, the fundamental unit of computational models that can help contextualize the data obtained from molecular methods. These models enable the construction of synthetic microbial communities that can provide predictions at a community level [22]. These represent quantitative methods that enable the formulation of hypothesis for experimental validation. The method of choice depends on the parameters to be explored. Some parameters include directionality (positive, negative, or neutral), reciprocity (unidirectional or bidirectional), strength, mode of action, and spatiotemporal variation (hours, weeks, order of colonization) [23, 24]. A combination of qualitative strategies and quantitative models will enable discovery of interdependencies in networks with several interconnected partners such as those in syntrophic communities [25]. Although frameworks exist in literature to describe microbial interactions, there are limitations in understanding the terminologies used for inferring the data. Data is often represented with a single parameter using graphical axes, which can sometimes be misleading about the interaction among the partners [26]. This hinders the understanding of the nuances crucial to describing how individual interactions affect a community and its functional specialization. Therefore, there exists an imminent and pertinent need to develop systematic and rigorous methods to study microbial interactions [27]. While there are several reviews that have explored the topic [28–30], most delve into mathematical equations for network inference with a focus on computational aspects of microbial network construction/elucidation [31–35]. There is a notable absence of a comprehensive review to pique scientific curiosity of empirical and theoretical scientists alike [36]. The current review provides an overview of the traditional and upcoming qualitative methods commonly employed in the field of microbiology with quantitative frameworks involving microbial network construction to infer microbial interactions with a discussion of the relevant analysis methods to choose based on design of the study. The aim is to provide scientists with the relevance and validity of microbial mathematical models, enabling an integration of experimental as well as modeling methodologies which will go a long way in furthering our understanding of microbial community dynamics, allowing for novel inventions in infection prognosis, diagnosis, and therapy.

## Qualitative Methods to Study Microbial Interactions

**Fig. 1:**
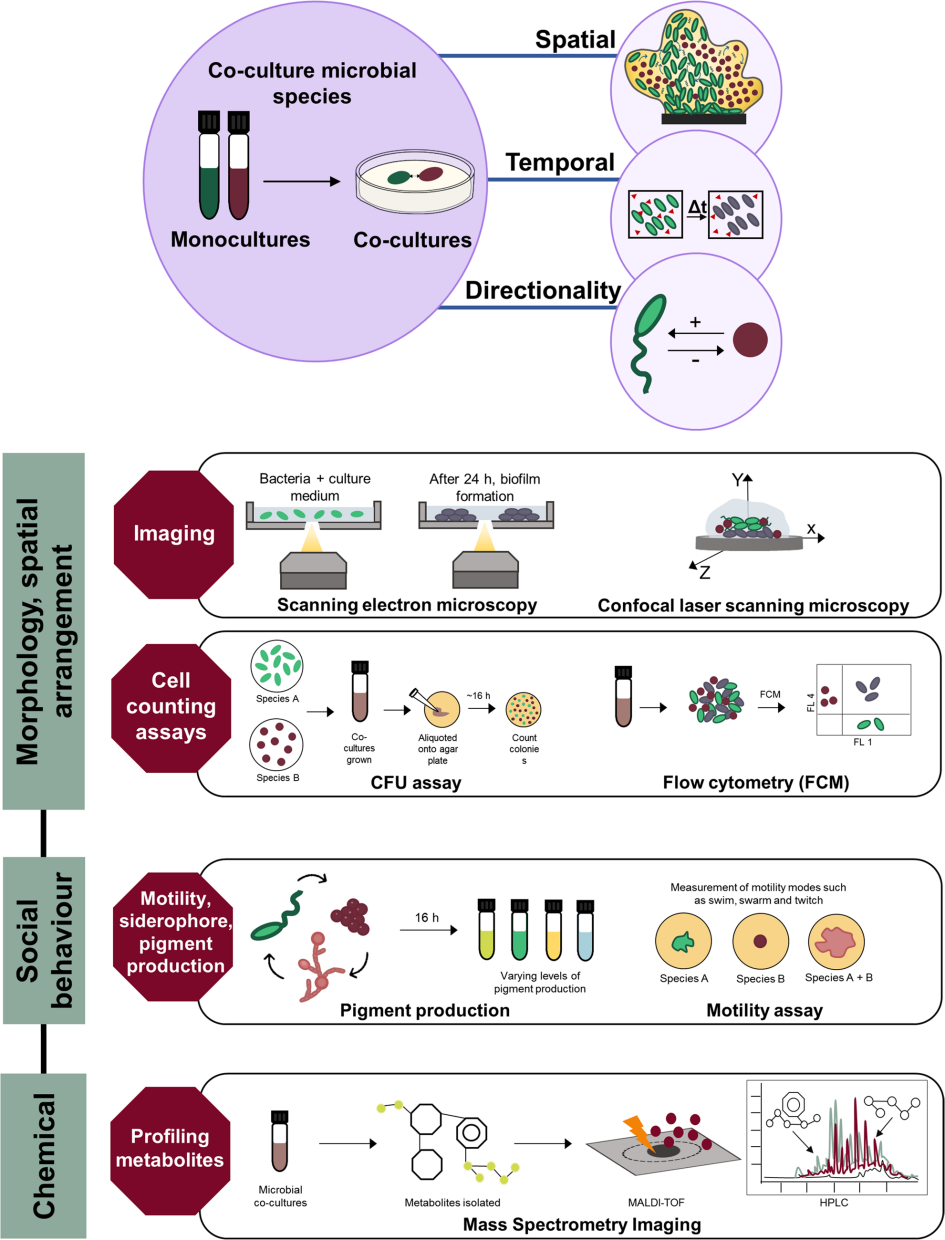
Overview of qualitative methods used to study microbial interactions

Qualitative assessment of microbial interactions involves determination of phenotypic changes such as morphology, spatial arrangement, metabolic activity, cross-fed metabolites, and quorum sensing (Table 1, Fig. 1).

**Table 1:** Summary of qualitative methods available to study microbial interactions with a description of the characterized microbial interactions/behavior

Phenotype	Method	Microbial interaction	Reference
Morphology			
Physical co-adherence	Fluorescence-based co-aggregation assay using two-chamber assay and PET membranes	Oral biofilms: *Candida albicans* co-localizes with *Fusobacterium nucleatum*	[37]
Colony morphology	Time-lapse imaging using MicrObial CHAmber (MOCHA) with double decker agar plates	Pellicle formation and colony morphology changes after release of extracellular DNA (eDNA) *Bacillus subtilis.* Novel colony morphology observed in *Bacillus amyloliquefaciens*	[38]
Mixed species biofilm structures	Scanning electron microscopy (SEM), transmission electron microscopy (TEM), confocal and fluorescence microscopy (CLSM)	Mixed biofilms of etiologic strains of *Aspergillus fumigatus* and *Staphylococcus aureus* isolated from infectious keratitis	[39]
Morphogenesis	IncuCyte time-lapse imaging and Neutrotrack (NT) analysis	Co-incubation of *Pseudomonas aeruginosa* with *Aspergillus fumigatus*: Siderophores pyoverdine and pyocyanin suppressed mycelial expansion of *A. fumigatus* in concentration dependent manner	[40]
Spatial arrangement			
Host microbial habitat	In vitro *Hydra* models on R2A agar plates to determine colony forming units (CFU) per individual	Microbiome of freshwater polyp *Hydra*: *Curvibacter* sp. strain AEP1.3 and *Duganella* sp. strain C1.2	[41]
Increased fitness and productivity	Biofilms cultured in three-channel flow chamber and visualized using time-lapse confocal microscopy	*Pseudomonas putida* and *Acinetobacter* sp.	[42]
Chemical compounds released			
Volatile compounds	Microbes cultured in nutrient limited agar followed by exposure to volatile compounds to assess difference in transcriptional response	Soil bacterium *Pseudomonas fluorescens* exposed to volatiles produced by soil co-inhabitants *Collimonas pratensis*, *Serratia plymuthica*, *Paenibacillus* sp., and *Pedobacter* sp.	[43]
Quorum sensing signals	Liquid chromatography-mass spectrometry–based metabolomic analysis	Metabolites produced by bacterial and fungal endophytes associated with brown algae (*Ascophyllum nodosum*, *Pelvetia canaliculata*, *Laminaria digitata*, and *Saccharina latissimi*) interferes with bacterial autoinducer-2 (quorum quenching)	[44]

### Co-culturing

Co-cultures provide a simple system to observe cell–cell interactions (direct and indirect), allowing for qualitative observation of directionality, mode of action and spatiotemporal variation. Cultivating microbial species together with the host provides an in vitro system to study host-microbe interactions that mimics in vivo conditions. Co-culture systems involving cell–cell contact include plating assays such as direct contact assay or mixed inoculum assays. Straight et al. [45] employed lawn cultures to study interactions between *Bacillus subtilis* and *Streptomyces coelicolor* which demonstrated an antagonistic interaction involving inhibition of aerial hyphae development and sporulation of *S*. *coelicolor*. McCully et al. [46] utilized plating assays to document interspecies social spreading between two soil bacteria, *Pseudomonas fluorescens* and *Pedobacter* isolates to gain mechanistic understanding of motility phenotypes generally exhibited by *Pseudomonas* spp. such as swimming, swarming and twitching. Antonic et al. [47] employed co-culture assay to study the effect of microbial interactions between *Pseudomonas aeruginosa* and *Staphylococcus aureus* on production of staphyloxanthin, a pigment produced by the latter known to be responsible for its virulence.

Co-culture systems to study contact independent interactions involving diffusible molecules using membrane-divided co-culture assay, conditioned media assay, or via microfluidics platform [48]. Membrane-divided co-cultures assay involves the physical separation of two microbial populations with semi-permeable membranes. Jo et al. [49] developed BioMe culture plate that offers higher throughput measurement of up to 30 pairwise interactions. Co-cultures can also be used to assess interkingdom contact–dependent interactions. Bor et al. [50] analyzed the interaction between predominant oral commensals *Fusobacterium nucleatum* and *Candida albicans* via a “two chamber assay” that utilized a polyester (PET) Transwell-Clear insert membrane. *F. nucleatum* cells were plated into the lower chambers of a 12-well plate. After placing 0.4-μm pore size membrane inserts, the upper chamber was filled with *C. albicans*. Membrane separation allowed for visualization of *C. albicans* hyphal cells without interference from *F. nucleatum* cells [51]. Conditioned media exchange involves growth of the interacting partner in spent growth medium of the other, which can reveal aspects of microbial interactions such as metabolic cross-feeding, metabolic exchange, nutrient availability, and release of metabolic byproducts [52].

While co-culture systems have been equipped with various modifications (membrane separation and microfluidics integration) to vastly improve its capability to capture microbial interactions, there exist several limitations. Setting up a mixed co-culture in laboratory conditions requires optimization at many levels especially when it involves interspecies interactions. Each microbial participant needs to be provided with optimal and uniform growth conditions, which includes catering to individual substratum and nutrient specificities [53]. Other optimization parameters include timing and ratio of inoculation of microbial partners and competition among partners. Poor experimental design leads to ineffective data acquisition and analysis at later interaction stages [54]. This can result in skewed experimental results, which cannot be used to extrapolate our knowledge of microbial interactions occurring within the system. Major limitation of pairwise co-culture methods lies in its ability to accurately reflect complex higher order interactions, those that are commonly found in natural microbial ecosystems. To obtain a more complete picture, co-culture techniques need to be integrated with experiments that can provide quantitative measures such as automated plate reader technology [49] and omics technologies [55]. The combined data can be used to build computational models that can be harnessed to build synthetic communities that can predict the nature of higher order interactions crucial to the functioning of the ecosystem [56].

### Imaging Technique to Study High Density Microbial Communities

Advancement in microscopy techniques such as scanning electron microscopy and confocal microscopy has allowed for the multidimensional imaging of complex 3D high-density microbial communities such as biofilms. Ramírez Granillo et al. [39] visualized monomicrobial and polymicrobial biofilms of microbes involved in infectious keratitis, *Aspergillus fumigatus* and *Staphylococcus aureus*. Differences in texture and distribution of extracellular matrix (ECM) were observed in mixed biofilms. Haagensen et al. [42] employed three channel flow chambers to study the dynamic interactions in the polymicrobial biofilm comprising of *Pseudomonas putida* and *Acinetobacter* sp. isolated from creosote-polluted aquifer. The setup using silicon tubes and microelectrodes ensured supply of oxygen and allowed dynamic measurement of oxygen concentration. The confocal images revealed that abundance of *Pseudomonas putida* were the highest within microcolonies of *Acinetobacter* sp.

### Mass Spectrometry

Mass spectrometry imaging (MSI) when integrated with genomic and transcriptomic studies provides precise information on chemical signaling and spatial profiles of complex microbial communities [57]. Additionally, metabolite exchanges, secondary metabolite production, and metabolic cross-feeding among microbial species can be visualized over space and time. Shih et al. [58] employed MSI to study bacterial cannibalism in *Bacillus subtilis* wherein the sporulating cells are known to kill the non-sporulating cells. Here, standard liquid co-cultures followed by quantification of metabolites could not be performed since the cannibalistic phenotype could only be observed on solid media which was not readily amenable for metabolic characterization. Therefore, researchers employed MSI wherein the culturing was done on a thin layer agar, photographed, exposed to a 1:1 mixture of acids (α-cyano-4-hydroxycinnamic acid and 2,5-dihydroxybenzoic acid), dried for three hours and subjected to matrix-assisted laser desorption/ionization-time of flight mass spectrometry imaging. This allowed for identification of two cannibalism-associated proteins produced by *B. subtilis* namely sporulation killing factor and sporulating delaying protein [58].

## Quantitative Network Models for Assessment of Microbial Interactions

**Fig. 2:**
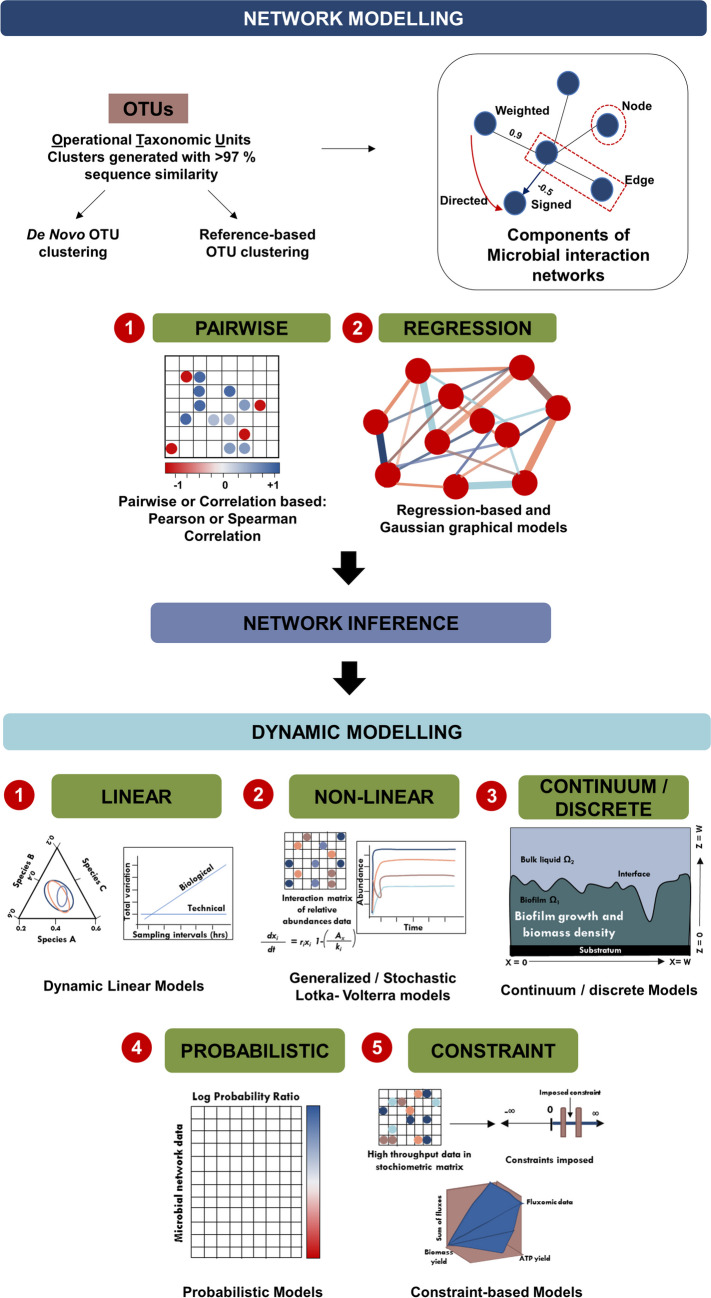
Overview of quantitative methods used to study microbial interactions

Since there remains a vast majority of microbes that cannot be cultured in a laboratory, the composition of the various species in the microbial community can be determined by procuring environmental DNA samples and conducting a next generation sequencing analysis. 16S metagenomics involves profiling the hypervariable regions of the bacterial taxonomic barcode gene coding for 16S rRNA [59]. Analogous to 16S sequencing, identification of fungal species involves sequencing the internal transcribed spacers along with gene coding for 5.8S rRNA (ITS1-5.8S-ITS2 region). Omics approaches are frequently used in microbial ecology for enumeration of microbes as operational taxonomic units (OTUs). However, this approach fails to test the causal relationships and microbial interactions that drive the microbiome’s structure and composition. [60]. Coupled with a quantitative framework, high throughput sequencing data can be harnessed to test significant species associations and make metabolic predictions of microbial behaviors. A fine example of the use of a quantitative framework to test for significant species associations driving microbial communities is demonstrated by Ontiveros et al. [60]. In this study, microbial populations thriving in extreme habitats such as in high altitude mountain lakes (Pyrenees) were profiled using 16S rRNA gene sequencing. Taxon abundances were then used to build co-occurrence networks using probabilistic methods. Significant co-exclusion and co-occurrence pairs along with the influence of relevant environmental parameters such as pH were tested with one-way ANOVA and chi square tests, providing means to identify significant associations that can be experimentally validated. It further follows that integrated experimental data obtained from multiple sources can be amplified and harnessed to yield meaningful insights into the functioning of microbial ecosystems with relatively inexpensive infrastructure albeit with significant technical expertise. This brings forth the need for understanding and developing quantitative computational models that can harness culture and omics data for inferring microbial interactions (Fig. 2).

### Networks and Network Inference

Networks lie at the heart of computational models and represent diversity in a microbial community. Associations between taxon abundances of microbes present may be hypothesized allowing for network inference [61]. Following this, correlations can be validated by direct evidence such as co-culture experiments and 16S metagenomics. Network inference–based approaches can be taken to decipher the ecological relevance of unknown taxon groups (“microbial dark matter”) towards community structure [62]. Transitioning from co-cultures to complex ecosystems is a progressively difficult task. One way to streamline the analysis would be to feed this data into statistical methods such as correlational and association analyses to analyze co-occurrence and dependence of different microbial species. This can be used to create a computational model that can be further used to develop better tools and predict new microbial interactions that can be harnessed to understand less studied microbial ecosystems.

Computational modeling involves the construction of network models (static or dynamic) and/or reconstruction of existing microbial networks or creation of new networks as a synthetic microbial consortium. For example, a typical metagenomics study analysis would involve preparing a list of different microbial species present in the community as OTUs with an associated quantitative measure of abundance in terms of the number of reads representing each OTU. Each attribute of the interacting partners such as time dependence, spatial dependence, site of interaction, habitat in which interaction takes place, and the predicted compounds involved based on the known metabolic capabilities of the microbes will be assigned a numerical value followed by network inference for further validation. Network inference with the abundance patterns can be quantified with pairwise (similarity-based) and complex (regression-based and rule-based) approaches [63].

#### Pairwise or Similarity-Based Network Inference

Pairwise or similarity-based network inference is used to evaluate the similarities between two interaction partners [64]. A quantifiable factor measures the similarities based on mutual exclusion of the interaction partners over multiple samples. All permutations based on the abundance data are then combined to construct the network. Freilich et al. [65] created an ecological pairwise network by mining central repositories of research such as PubMed for pairwise interactions. Researchers obtained annotations for all the bacterial species whose complete sequences were available and queried the database for each pairwise interaction. The number of abstracts that matched the query were counted followed by network construction such that networks of species forms “nodes” and the interactions between microbial species were termed as “edges”. Ecological parameters used to characterize the different clusters included maximal growth rate, respiration mode, and competition level for natural resources. With these methods, researchers were able to plot the first complex ecological model of bacterial interactions.

#### Complex/Regression or Rule-Based Network Inference

Natural/synthetic microbial communities generally cannot be reduced to simple pairwise interactions. Such an approach affects the resolution of higher-order interactions and hinders classification of interactions affected by abiotic and biotic factors. Inference of networks with a multidimensional approach can provide insights into the nature of interaction, such as the fitness or metabolic cost, spatiotemporal dynamics, and several other parameters that determine the ecological outcome [27]. Regression-based network inference involves predicting relationships between dependent (abundance of target species) and independent variables (abiotic and biotic factors). Here, network inference from multivariate linear regression models [66] incorporates environmental traits as additional factors, allowing us to predict relationships between species and environmental traits [61]. The statistical techniques used for analysis include Pearson or Spearman correlation and local similarity analysis for procuring abundance data. For presence–absence data, Fischer’s exact test is used to determine hypergeometric distribution. Rule-based network inference involves listing all possible combinations of taxa and then generating feasible rules for each set of taxa. Complex interactions are depicted as directed hypergraphs which represent edges connecting two nodes (hyper edge; three nodes).

### Dynamic Modeling

Static networks when complemented with dynamic models capture the dynamics and stabilities of microbiomes, giving a biological perspective of microbial interactions (Table 2). Static methods analyzing co-variances of the abundances of each microbe can efficiently determine whether positive or negative correlations exist between the different taxa within a microbiome. However, they fail to point out the bi-directionality of interactions and the underlying mechanisms behind taxon abundance patterns observed in a microbial community [67]. Hence, construction of dynamic models based on static networks provides improved and reliable network inference. These models predict the directionality and map time-dependent properties mediated by factors such as environmental fluctuations, which can operate over spatial–temporal scales [68]. Stein et al. [69] employed dynamic ecological models to study stability of intestinal microbiota following administration of antibiotic clindamycin. Using non-linear first order ordinary differential equations (ODE) based model which considers ecological time resolved data, the authors were able to observe the differential composition of microbial communities within their treatment groups. Data obtained were described using a set of ODE or Boolean functions which contained variables attributed to all factors that could play a role in mapping the interactions. Similarly, high throughput data obtained from qualitative laboratory culture techniques can be described with these equations [70]. Dynamic models are often used to infer synthetic microbial consortia. They allow for rigorous and reproducible analysis of microbial interactions by reducing environmental heterogeneities common in natural higher order interactions [71]. When encountering a spatially constructed environment, partial differential equations can be used for network inference. These models can be linear, non-linear, discrete, continuous, probabilistic or constraint-based models [3].

**Table 2:** Summary of quantitative frameworks commonly employed to study microbial interactions with a brief discussion on its implementation, strengths and limitations

Microbial model	Method/use-case	Tools for implementation	Strengths	Weaknesses	References
Linear model	**Dynamic linear models (multinomial logistic normal dynamic linear models—MALLARD framework):** To investigate the effect of technical variation in ex vivo studies of human gut microbiota. Model was constructed with taxon abundances at family level obtained by 16S rRNA sequencing	R packages *shapes*, *ape*, *ks*, and* philr*	Efficiently accounts for problems commonly observed with longitudinal time series analysis of microbiome studies such as missing data, count variability and other factors that cause technical variations	Being a model that uses log ratio transformation to describe dynamics, it is computationally intensive. In addition, MALLARD frameworks are unable to distinguish between the true absence of an OTU from one that is absent because it did not meet the threshold detection criteria. In some cases, this can influence community dynamics	[72, 73]
Non-linear model	**Generalized Lotka-Volterra (gLV) model:** To study microbial community assembly dynamics in a synthetic gut microbiome model. Model was constructed with relative abundances obtained from 16S rRNA sequencing and absolute abundances obtained from simultaneous OD_600_ measurements	Ordinary differential equations involving number of species, growth rate and intra/inter species interaction coefficients were analyzed with MATLAB (MathWorks)	Reliable model for bottom-up analysis of microbial communities dominated by pairwise interactions	Being a phenomenological model, it may not be able to incorporate higher order, complex metabolic interactions and environmental heterogeneous gradients. Model needs to assume intraspecies interaction strength to be null which is not always true	[74]
	**Stochastic gLV model (extended Kalman Filter algorithm):** A proposed improvement on the gLV model to better understand microbial interactions for designing antibiotic/probiotic therapies in infectious diseases such as *C. difficile* infections	Matlab 8.3.0	True positive, true negative and false positive interactions are recorded. Sensitivity and specificity analyses help build an accurate topology of the simulated microbial network	Captures only pairwise species relationships, incapable of extending to complex microbiomes	[75]
Continuum model	To study oligotrophic biofilm dynamics such as the influence of cationic metals and ionic strength on the attachment of bacterial cells to the substrate (distribution pipelines transporting potable water)	MATLAB	Descriptive model accounting for several parameters and one of the first to model the influence of metals, i.e., ionic strength on bacterial attachment	Behavioral properties of bio-specific processes can be included to further understanding of biofilm growth	[76]
Discrete model	**Cellular automaton:** To study spatiotemporal heterogeneity in microbial communities	C# on the Unity platform	Octahedron grid geometry in 3D model to allow spatiotemporal study of biofilm growth by simulating self-similar evolution in bacterial biofilms	Model contains several parameters limiting simultaneous variation	[77]
	**Genome scale metabolic model:** To understand quorum sensing and spatial patterning using synthetic simulations	MATLAB R2019b (Mathworks) – picCASO package	Offers significantly reduced computational complexity to study large microbial communities and accounts for factors such as spatial heterogeneity in synthetic consortia	Currently limited by quantitative measurements available to study aspects of natural microbial communities such as intercellular dynamics and temporal gene expression	[78]
Probabilistic model	**Bayesian network model:** Popular models for modeling gut microbiome dynamics to predict microbial networks of clinical relevance. Dynamic Bayesian network (DBN) models enable study of complex networks with feedback pathways. DBN models are also ideal to explore longitudinal multi-omics datasets	MATLAB (Mathworks) – CGBayesNets package	Model of choice for studying complex higher order microbial interactions	The underlying mechanisms that contribute to predicted relationships might not be clearly understood	[79]
	**Bayesian network model for specific analysis of integrated longitudinal multi-omic datasets**	Python, MATLAB	Trained model combines taxon, host genes, bacterial genes and metabolites data to form temporal networks	DBN requires in silico validation as network edges do not imply causal relationships	[80]
Constraint model	**Microbiome Modeling Toolbox (MMT)—constraint-based reconstruction and analysis framework (COBRA)**: It allows visualizing microbe-microbe and host-microbe interactions	Microbiome Modeling Toolbox—MATLAB (Mathworks)	Relatively user friendly model for the analysis of microbe-microbe and host-microbe metabolic interactions	Computationally intensive. However, a version update reportedly has significantly reduced computation times by adopting parallelization	[81, 82]
	**COBRA model with flux balance analysis:** Novel use of constraint based microbial community modeling on an individual with episodic inflammation of GI tract	COBRA and Microbiome Modeling Toolbox—MATLAB (Mathworks)	Inclusion of flux balance analysis allowed for the adoption of the COBRA framework for understanding net metabolite production as a function of changes in gut microbiota composition	Does not account for inclusion of confounding factors such as antibiotic use. However, the model can integrate metagenomic and metabolite data efficiently to generate novel hypothesis for testing	[83]

#### Linear models

Dynamic linear models (DLMs) analyze auto-correlated time series data sets using Bayesian approach and are commonly used to describe the robustness of a microbial community [34]. DLMs are usually used in its multivariate form since it takes into account the co-dependencies between different variables [84]. Silverman et al. [73] extended DLMs to a multinominal logistic normal model to study artificial human gut microbiota. A continuous flow anaerobic bioreactor system was constructed that functioned as an ex vivo artificial human gut microbiota model. DLM coupled with generalized dynamic linear models was used for time series data modeling framework (MALLARD). This system allowed for the characterization of the impact played by technical sources of variation observed in ex vivo experiments, specifically in artificial human gut.

#### Non-linear Lotka Volterra Model

Choice of the mathematical equation/model is crucial to the interpretation of the model. Most popular models are non-linear Lotka Volterra (LV) models that were first employed to describe predator–prey relationships. Simple systems involving a maximum of two species can be captured, making these models relevant for competitive interactions. Using equations to reflect on magnitude of the influence of interactions on the fitness of an organism without considering any other aspect of the interaction, these models are an attractive and appropriate choice to study pairwise interactions. Shibasaki and Mitri [85] employed LV models to assess the stability and spatial dynamics of a gut microbiome community and found that stability of the downstream communities was improved by enhancing positive interactions in the upper communities. However, the classic LV model fails to capture the diversity of pairwise interactions in cases where additivity cannot be assumed. They often fail to capture ecologically relevant aspects of microbial interactions and need to be supplemented with in vitro experiments to assess the applicability of the model [86]. Hence, classic LV models cannot be used to model multispecies interactions [87]. The generalized Lotka-Voltera (gLV) model is an extension of the logistic growth model and can represent any number of species using absolute abundances data [30]. It is inclusive of interactions such as neutralism, competition, and cooperation. Venturelli et al. [74] used gLV model to assess the gut microbiome dynamics by measuring relative abundances of species as a function of time for pairwise interactions. Alshawaqfeh et al. [75] took this further with the addition of a noise term to compensate for uncertainties in dynamics along with reduced computational time and other modeling errors. A GUI-based interactive platform was developed for gLV-based modeling providing mathematical models using the temporal microbial abundance data [88].

#### Continuum/Discrete Models

Microbial communities with dense biomass and an abundance of physico-chemical and biological processes, such as biofilms, can be represented by dynamic continuum [76] and/or discrete models. Both these models investigate how the biomass spreads and diffuses into the external environment. Discrete models (bottom up approach) [77] represent the interactions between microbes present in the biomass with their surroundings [89]. Discrete models include cellular automaton models and individual-based models (IBM). Cellular automaton simulation represents individual cells in biofilms as discrete units that are then dynamically rearranged to provide detailed simulations of biofilm morphologies [90]. IBM models of biofilm represent bacterial cells as rigid spheres and are described with several parameters (phenotype, position, mass, volume, velocity, growth rate). Jayathilake et al. [91] coupled IBM with large scale atomic/molecular massively parallel simulator (LAMMPS) and used this integrated model to detect both biological and physical processes that take place in a biofilm during its formation, detachment, and deformation under different environmental conditions.

#### Probabilistic Models

Probabilistic models allow for measurement of uncertainty in higher order interaction networks with minimal bias using probability theory [92]. Dynamic Bayesian networks (DBN) is an example of a probabilistic graphical model, frequently used to capture temporal changes in clinical and ecological settings. McGeachie et al. [79] utilized DBN analysis to investigate progression of colonizing microbiota in infant, revealing Bacilli as initial colonizers (facultative anaerobes), temporarily out-competed by Gamma-proteobacteria following colonization by *Clostridia* (obligate anaerobes). DBN are usually used for modeling metagenomic data from microbial communities, but they can only analyze a single set of omics data. To overcome this obstacle, Ruiz-Perez et al. [80] developed a pipeline for analysis of longitudinal multi-omics data. This workflow proceeds by first aligning multi-omics data followed by usage of DBN to reconstruct the model. Probabilistic topic models allow prediction of microbial dynamics in individuals by obtaining weight of each OTU in gut microbiome [93].

#### Constraint-Based Models

To derive the best possible representation of the interactions within a microbial community, it is important to cross-link different data types with appropriate computational biology techniques. One such approach is constraint-based reconstruction and analysis (COBRA). This analysis method has the advantage of integrating with in vivo and in vitro models which can present the structure and function of the microbiome [94]. It is generally used to map interaction networks involving genes involved in metabolism, regulation of transcription and various biochemical functions [95]. Baldini et al. [81] developed a microbiome modeling toolbox for analysis of pairwise microbe–microbe and microbe–host interactions using constraint-based modeling. Basile et al. [83] employed constraint-based models to study variations in gut microbiome ecology over 6 months in a single individual affected with inflammation of gastrointestinal tract. With the help of the model, researchers were able to identify time-correlated microbe-metabolite networks shaping the dynamic disease state.

### Pitfalls for Network Models

Obtaining microbial abundance data from microbial taxa from “rare biospheres” can cause data sparsity problems. Carr et al. [96] elucidated how correlational data cannot predict unreported interactions and often reflect true interactions only for a very narrow range of conditions. Choice of mathematical equations remains a key factor in the assessment of the dynamic models influencing network inference. Differential equations used in ecological models often procure absolute abundances as the only variable, limiting model’s capability to study interactions in different environments. Researchers have incorporated modifications accordingly to consider the molecular interactions and cellular dynamics that would describe the metabolic state of the cell. However, it remains a challenge to accurately describe the substrates used, products formed, and tracking of the corresponding biochemical responses in a co-culture experiment.

## Current Challenges in Bridging Theoretical and Experimental Data

Theoretical microbial models present an attractive method to visualize, predict, and validate experimentally observed microbial interactions [32]. For example, computational modeling of taxon abundances obtained via high throughput sequencing in combination with omics data on metabolic status allows for capturing unbiased dimensionality of microbial interactions. Mathematical models often help complement conventional experimental studies and in vitro models [77]. However, while one can agree that integrating theoretical models with experimental data can mutually help both enhance the predictive capabilities of the experimental data as well as provide validation to the computational model, it is debatable whether such a correlation has been observed in practice. There exist several reports wherein computational models have been experimentally validated [66, 68, 75, 80]. For example, Ruiz-Perez et al. [80] developed a bioinformatics pipeline to enhance the capabilities of DBN networks by allowing for integration of longitudinal data obtained from a variety of omics technologies including metabolomics and metatranscriptomics. Briefly, the pipeline begins with normalization of taxon abundance data extracted from metagenomic sequencing studies. This is followed by “spline interpolation” of time versus abundance data to obtain a “best fit” curve to correct for external factors such as missing time points. The application of mathematical functions to study bacterial communities were described as early as 1976 when they were introduced to study bacterial motility using information from photon correlation spectroscopy experiments. A key feature of splines is it allows for ready integration of multiple data points as “constraint” parameters allowing to achieve the best fit of approximation data to experimentally observed data. This significantly enhances the validity of each predicted data point [97]. Ruiz-Perez et al. [80] tested the in silico metabolite taxon edges/predictions in organisms such as *Pseudomonas aeruginosa* and *Escherichia coli* and found a positive correlation between theoretical and experimental data. However, inference of microbial interactions from predicted co-occurrence networks can be sometimes erroneous [98]. Microbial communities are often found in heterogeneous environments with a gradient of factors contributing to its highly varied ecological interactions. Simple co-occurrence networks from computational models that are insufficiently trained to capture the directionality and causality of complex and dynamic interactions will be significantly limited in its predictions [99]. Therefore, while positive correlations might corroborate the reliability of the model, lack of experimental validation for a predicted interaction might not mean that the prediction is necessarily wrong but only that certain critical parameters might be missing.

Microbes are rarely solitary in nature, often found engaged in highly heterogeneous networks as communities. A common reason for lack of correlation between the model prediction and observed data is because community phenotypes are modeled based on individual phenotypes, a problem that is prevalent even in inferences made from qualitative experimental methodologies [100]. Models often trade between optimizing individual species’ fitness and the entire community’s fitness function [26], leading to inaccurate assumptions about the super-optimal behavior of members in the community. Metabolic networks are often mathematically analyzed using flux balance analysis (FBA) [101]. FBA allows for prediction of microbial growth rate and production rate of metabolites of interest. Traditional genome scale metabolic models assume instantaneous biomass maximization of all members of a microbial community (community-level objective function) [102]. However, this approach is flawed since microbes, similar to higher order organisms, display varied phenotypes, as a result of several decisions influenced by the community with known rate-yield trade-offs where maximum biomass production may not be the desired goal [103, 104]. Hence, prediction of a microbial community’s emergent properties remains a challenge in systems biology [102]. Some models that have been introduced to overcome this include the optCom model [100] which is a constraint based modeling approach. This model has been used to explain syntrophic interactions involving transfer of a key metabolite as well as complex interactions of phototrophic microbial mats found in Yellowstone National Park. While existing models may be modified with additional parameters/constraints and appropriate data acquisition to predict different facets of microbial interactions, an integrational gap exists between experimental data and computational models [36]. So far, prediction tools have been validated with corresponding high throughput data and experimental models. However, extending the horizon towards predicting unknown interactions between taxons remains to be studied.”

## Conclusion

Microbial interactions remain the driving force behind the establishment and maintenance of a microbial population. Extensive efforts have been put towards obtaining microbial abundance data from different communities existing in different ecological states to build reliable microbial computational models. With the advent of machine learning and deep learning algorithms, adoption of quantitative frameworks to study microbial studies has increasingly become more accessible and feasible [105]. However, there still exits several limitations in distinguishing true microbial interactions and non-random process or casual relationships. An understanding of how microbial communities assemble and how this translates to their functionalization and ecological outcome has vast potential applications in avenues of medicine, agriculture, bioprocessing, and food industries [106]. Potential to engineer metabolic network models comprising of commercially important microbial species remains unfulfilled. Standardized adaptation of qualitative and quantitative methods can enable mechanistic and reproducible exploration of microbial interactions and provide relevant information on key molecules, interactions, and strategies to better understand polymicrobial communities and better the health of plant, animal, environment, and human alike.

## Data Availability

Not applicable.
